# Nephrocystins play a crucial role in renal epithelial morphogenesis via the regulation of Wnt/PCP components Dishevelled and Rho GTPases

**DOI:** 10.1186/2046-2530-1-S1-P100

**Published:** 2012-11-16

**Authors:** S Saunier, HM Gaudé, R Montjean, F Silbermann, V Grampa, C Burcklé, E Montenont, M Delous, C Vesque, C Jeanpierre, C Antignac, F Terzi, S Schneider-Maunoury

**Affiliations:** 1 Hôpital Necker, Inserm U983, France; 2 CNRS UMR 7622, INSERM U969, France; 3 INSERM U845, France

## 

Nephronophthisis, a hereditary nephropathy characterized by interstitial fibrosis and cyst formation, is caused by mutations in NPHP genes encoding the ciliary proteins called nephrocystins. We investigate the function of nephrocystin-1, -4 and -8, *in vitro* and *in vivo* in mammalian kidney cells and in zebrafish respectively. Depletion of either *NPHP1* (N1-KD), *NPHP4* (N4-KD) or *NPHP8* (N8-KD) by shRNA-mediated knockdown in MDCK cells led to abnormal ciliogenesis and epithelial morphogenesis defects in 3D culture. Moreover nephrocystin-4 modulates the Wnt pathways during morphogenesis of the zebrafish pronephros and in vitro, via proteasomal degradation of cytoplasmic/membranous dishevelled. In addition, we demonstrate that nephrocystin-8 is required for dishevelled stability at the basal body essential for proper PCP. In either N1-KD or N4-KD cells, we also showed an over activation of Cdc42 and RhoA, downstream targets of dishevelled. This was accompanied by actin cytoskeletal disorganization, enhanced spreading on collagen, over-activation of proteins that regulate focal adhesion structures i.e p130cas-Pyk2 and increased cell migration. Interestingly, the stable expression of dominant negative form of Cdc42 in knockdown cells rescued the migration and the 3D phenotypes. In parallel, we observed that loss of Nphp4 in mice caused cystic tubular dilatation after subtotal nephrectomy correlated with alteration of ciliogenesis and over activation of Cdc42 and RhoA. Our data show a role of nephrocystins in epithelial cell organization and kidney morphogenesis via the regulation of the Wnt/PCP components including dishevelled and the Rho GTPases.

